# Associations Between Physical Function and Physical Activity in Individuals With Chronic Ankle Instability: An Exploratory Prospective Study

**DOI:** 10.1155/tsm2/1557781

**Published:** 2026-08-10

**Authors:** Ji Yeon Choi, Colleen M. Vogel, Michaela A. Schenkelberg, Christopher J. Burcal, Adam B. Rosen

**Affiliations:** ^1^ School of Health and Kinesiology, University of Nebraska Omaha, Omaha, USA, unomaha.edu

**Keywords:** balance, chronic ankle instability, dorsiflexion strength, physical activity, range of motion, sedentary behavior

## Abstract

**Background:**

Individuals with chronic ankle instability (CAI) often exhibit decreased physical activity (PA) which may increase sedentary behavior and chronic disease risk. However, factors contributing to decreased PA in individuals with CAI remain unclear. This study aimed to explore associations between physical function and PA in individuals with CAI.

**Methods:**

Thirty individuals with CAI participated and completed clinical assessments of range of motion (ROM), strength, balance, and functional performance. PA was monitored for 4 weeks using a tracker. Pearson correlations were used to examine associations between physical function and PA, followed by backward selection linear regression to identify the best multifactorial model predicting PA.

**Results:**

Distance walked and floors climbed were associated with eversion ROM (*β* = −0.42, *p* = 0.02; *β* = −0.40, *p* = 0.03, respectively). Sedentary minutes were associated with WBLT (*β* = −0.58, *p* < 0.001). Light activity minutes were associated with dorsiflexion strength (*β* = 0.39, *p* = 0.04). Very active minutes were associated with balance (*β* = −0.50, *p* < 0.01) and eversion ROM (*β* = −0.35, *p* = 0.04). Number of calories burned was associated with inversion ROM (*β* = −0.38, *p* = 0.03).

**Conclusion:**

This study suggests that eversion ROM, inversion ROM, WBLT, balance, and dorsiflexion strength may be associated with aspects of PA in individuals with CAI. Rehabilitation programs may consider these factors to support PA.

## 1. Introduction

Lateral ankle sprain (LAS) is one of the most common lower extremity musculoskeletal injuries in physically active individuals, with approximately 2 million cases occurring annually in the United States [1, 2]. LAS has a high recurrence rate and can lead to persistent symptoms and reduced ankle function [3, 4]. Due to its high prevalence, LAS represents a significant public health concern, contributing to work and leisure time loss, reduced physical activity (PA), and long‐term disability [3, 5]. The annual direct and indirect costs attributed to ankle sprains in the United States can exceed $6.2 billion [3].

Up to 40% of individuals following an initial LAS develop chronic ankle instability (CAI), characterized by recurrent sprains, repeated giving‐way episodes, functional limitations, and ongoing symptoms such as pain, weakness, limited ankle range of motion (ROM), and decreased self‐reported function [6, 7]. Previous studies in adult populations consistently indicate that individuals with CAI demonstrate reduced PA, including fewer daily steps, lower self‐reported PA, decreased cardiorespiratory fitness, and higher body fat percentage compared to those without CAI [8, 9]. Furthermore, individuals with a history of multiple ankle sprains also tend to have higher body mass and self‐reported disability compared to those with a single sprain [10].

Reduced PA can lead to sedentary behavior and increase the risk of chronic diseases [8, 11]. Ankle function plays a critical role in basic movements and daily activities such as standing, walking, running, and jumping, which are necessary for both daily living and recreational activities [12]. However, it remains unclear which specific functional limitations contribute to PA in this population. Understanding the factors influencing reduced PA in these individuals can help identify opportunities for rehabilitation and strategies to prevent physical inactivity, which may subsequently lower joint health issues and associated conditions. Therefore, the purpose of this study was to explore associations between physical function and PA in individuals with CAI.

## 2. Materials and Methods

### 2.1. Participants

This study was designed as an exploratory analysis, and no formal a priori sample size calculation was performed for the current analyses. This study was approved by the University of Nebraska Medical Center Institutional Review Board (0008‐20‐EP; initial approval date 2/14/2020), and all participants completed informed consent prior to participating. A total of 32 individuals with CAI were enrolled from a local midwestern community, ranging in age from 19 to 35 years. Thirty participants completed the study and were included in the final analyses. Participants were included if they (1) had a history of at least 1 significant ankle sprain, with the initial sprain occurring at least 12 months prior to enrollment; (2) have experienced the most recent injury more than three months prior to study enrollment; (3) reported episodes of “giving‐way,” recurrent ankle sprains, or feelings of instability; (4) have self‐reported ankle instability scores of ≤ 25 on the CAIT, as previous studies have supported this cutoff based on recalibration analyses [13, 14]. Exclusion criteria for participants in this study were those with (1) a history of previous surgery to the musculoskeletal structures; (2) a history of fracture in the lower extremity requiring realignment; (3) a history of acute injury to the musculoskeletal structures that affected the function of the joints, resulting in at least one day interruption to the desired PA during the previous 3 months [15].

### 2.2. Procedures

#### 2.2.1. Overview

Prior to participation, each participant completed human subjects review board–approved informed consent documentation. Participants also completed an ankle injury history questionnaire and Cumberland Ankle Instability Tool (CAIT) [16]. We measured baseline clinical assessments of the ROM, strength, balance, and functional performance. After baseline testing, participants were provided a PA tracker (Fitbit Versa 2, Fitbit Inc., San Francisco, CA, USA) to track their PA for 4 weeks. Participants were asked to create a personal account on the Fitbit website. They synchronized the device with their personal smartphone and shared their login information with the researchers. Participants were instructed to always wear the PA tracker on their wrist except while charging the device. Text messages were sent to participants twice a week, every Monday and Thursday, to encourage continued wear of the PA tracker [17].

#### 2.2.2. Patient‐Reported Outcomes

All participants completed an ankle injury history questionnaire that included questions related to demographics, injury history, ankle “giving‐way” episodes, and PA participation. Participants then completed the CAIT patient‐reported outcome (PRO) measure to assess their perception of their physical function and disability: The CAIT is a disease‐specific PRO consisting of nine items intended as a screening tool and provides information regarding the severity of CAI [16]. Lower CAIT scores indicate greater perceived ankle instability.

#### 2.2.3. Clinical Assessments

*ROM:* Ankle ROM was assessed using a standard goniometer to measure dorsiflexion, plantarflexion, rearfoot inversion, and rearfoot eversion [18]. Active ROM was assessed, and participants were instructed to move the ankle through their maximum available ROM in each direction. Dorsiflexion and plantarflexion ROM were measured under nonweight‐bearing conditions with the knee flexed at 90° in a seated position to minimize gastrocnemius contribution. For dorsiflexion and plantarflexion, the fulcrum was positioned at the lateral malleolus, the stationary arm aligned with the fibular shaft, and the moving arm parallel to the fifth metatarsal. Rearfoot inversion and eversion ROM were assessed with participants in a prone position with the knee fully extended and the foot positioned over the edge of the table. The neutral position was defined as 0°, with the calcaneus aligned vertically relative to the lower leg. The fulcrum was positioned at the midpoint between the medial and lateral malleoli, the stationary arm was aligned with the midline of the lower leg, and the moving arm was positioned at the midline of the calcaneus. Participants were instructed to actively move into inversion and eversion to their maximum available range, and the maximal end‐range angle was recorded. The weight‐bearing lunge test (WBLT) was used to assess closed‐chain dorsiflexion ROM. During this test, participants lunged forward toward a wall while maintaining heel contact with the ground [19]. The angle of tibia relative to vertical was measured using an inclinometer placed along the anterior aspect of the tibia. Three trials were performed for each measure, and the average value was used for analysis.

*Strength:* A hand‐held dynamometer was used to measure the ankle strength of the dorsiflexion, inversion, and eversion in a seated position with the knee flexed to 90°. Plantarflexion strength was not assessed due to limitations of hand‐held dynamometry in measuring high‐force muscle contractions [20]. For each muscle group, the dynamometer was placed on the distal segment of the foot corresponding to the direction of force application (dorsal aspect for dorsiflexion, medial aspect for inversion, and lateral aspect for eversion). Participants were asked to perform three maximal voluntary contractions for each direction [18]. Participants were instructed to perform a ramp contraction by gradually increasing force over 1–3 s and then maintaining a maximal isometric contraction for 2 s. A 30‐s rest period was provided between trials to minimize fatigue. Standardized instructions were provided to minimize compensatory movement from proximal joints. The mean of the three peak values was used for analysis. Strength values were normalized to body weight and expressed as a percentage of body weight.

*Balance:* Balance was assessed via the modified balance error scoring system (mBESS) [21] with the Sway Mobile Balance Application (SWAY App), which utilizes a smartphone accelerometer to assess static balance. Participants held a smartphone at the center of their chest and performed five stance conditions: double‐leg stance, tandem stance with the right foot in front, tandem stance with the left foot in front, single leg stance with the right foot, and single leg stance with the left foot. The application calculates a composite static balance score across the five conditions based on the excessive movement of the center of mass.

*Functional performance testing:* The side‐hop test was performed to assess ankle function by hopping laterally on a limb over a distance of 30 cm [22]. Each repetition consisted of hopping laterally and returning to the standing position. Participants were asked to complete 10 repetitions as fast as they could. To measure the time, a smartphone stopwatch was used to measure the fastest time, and the average of the three trials was used for analysis.

*PA:* The PA outcomes of interest were step counts, the distance walked, the number of calories burned, and the number of activity calories, sedentary minutes, and minutes spent in light active, fairly active, and very active. This PA tracker uses a 3‐axis accelerometer to estimate the duration and intensity of activity, and GPS data were used to calculate distance walked [23]. Activity intensity categories were derived from the wearable device. Based on previous studies using data from Fitbit, activity intensity was interpreted using metabolic equivalent task (MET) calculations: sedentary (< 1.5 METs), light (1.5–3 METs), moderate (3–6 METs), and vigorous (≥ 6 METs) [23]. The number of calories burned was estimated by combining activity data with the basal metabolic rate (BMR), which represents the calories required to maintain vital body function at rest. Activity calories were estimated based on calories expended during physical activities [23].

### 2.3. Data and Statistical Analysis

The measures of injury history, self‐reported function/disability, ROM, strength, balance, and functional performance testing were the primary physical function outcomes of interest. PA variables were calculated using the 21 days of monitoring. The initial week of data collection was excluded to allow participants to acclimate to the PA tracker [18]. The PA tracker did not provide information on daily wear time, and nonwear days were identified as days with zero values across activity variables (e.g., steps, distance, and floors) and were excluded. For each participant, average daily values were calculated using all available days with nonzero activity. Pearson’s product moment correlations were performed between their PA and their clinical measurements, and the clinical measurements with the greatest significant correlations with their PA were used to identify the clinical factors most strongly associated with PA outcomes. A backward selection linear regression was used to determine the best fit multifactorial model to predict their PA outcomes. Models were selected based on statistical significance while minimizing the number of predictors. Additionally, multicollinearity was assessed using variance inflation factors (VIFs) and tolerance values. The significance level was set a priori at *p* < 0.05 for all statistical tests. All statistical analyses were conducted using IBM SPSS software (Version 31.0, IBM, Inc., Armonk, NY).

## 3. Results

One participant failed to wear the Fitbit during the study, and another participant’s data were not successfully synchronized to the account. Consequently, PA data from 30 of the 32 enrolled participants were included in the final analyses. Demographic data and descriptive statistics for the participants can be found in Table 1. Initial bivariate correlation analyses identified several associations between physical function and PA outcomes. Eversion ROM was associated with steps, distance, the number of activity calories burned, and the total number of calories burned. WBLT was associated with sedentary minutes. Dorsiflexion strength was associated with light active minutes, whereas inversion ROM was associated with fairly active minutes. Balance and inversion ROM were associated with very active minutes. CAIT was associated with the number of activity calories burned and the total number of calories burned. In addition, inversion ROM was associated with the number of activity calories burned and the total number of calories burned. Side‐hop performance was associated with floors climbed (Table 2).

**TABLE 1:** Mean (SD) and range of demographics data.

	Mean (SD)	Range
Gender (male/female)	16/14	
Age, years	22.50 (2.69)	19.00–35.00
Height, cm	173.47 (9.34)	152.50–190.00
Weight, kg	78.19 (15.85)	48.00–102.50
BMI	25.90 (4.56)	17.63–36.11
CAIT	17.40 (4.87)	7.00–25.00
WBLT, degree	42.19 (7.25)	21.70–60.60
EVS, %BW	8.08 (2.72)	4.16–15.61
IVS, %BW	8.29 (2.78)	3.76–13.92
DFS, %BW	15.84 (4.59)	9.39–27.59
mBESS	75.11 (17.26)	29.32–94.70
EVROM, degree	5.33 (1.79)	3.00–11.00
IVROM, degree	16.10 (5.57)	7.00–28.00
PFROM, degree	55.77 (9.60)	27.00–75.00
DFROM, degree	10.43 (5.61)	1.00–24.00
Side‐hop, sec	11.48 (4.67)	6.59–26.18
NC, kcal/day	2891.06 (748.11)	1560.29–4453.24
Steps/day	9085.72 (3643.55)	3551.83–18288.24
Distance, mi/day	4.18 (1.72)	1.48–8.46
SM, min/day	703.80 (152.12)	488.00–1256.43
LAM, min/day	250.12 (73.16)	135.17–392.41
FAM, min/day	26.53 (22.91)	2.14–96.38
VAM, min/day	25.99 (22.39)	0.90–84.24
AC, kcal	1445.87 (617.22)	396.44–3036.12

*Note:* EVS, eversion strength; IVS, inversion strength; DFS, dorsiflexion strength; EVROM, eversion range of motion; IVROM, inversion range of motion; PFROM, plantarflexion range of motion; DFROM, dorsiflexion range of motion; NC, number of calories burned.

Abbreviations: AC, activity calories; BMI, body mass index; CAIT, cumberland ankle instability tool; FAM, fairly active minutes; LAM, light active minutes; mBESS, modified balance error scoring system; SM, sedentary minutes; VAM, very active minutes; WBLT, weight‐bearing lunge test.

**TABLE 2:** Correlation coefficient (*r*(*p*)) between clinical measurements and physical activity outcomes.

	CAIT	WBLT	DFS	mBESS	EVROM	IVROM	Side hop
Steps	0.18 (0.17)	0.13 (0.25)	−0.18 (0.18)	−0.08 (0.34)	**−0.34 (0.035)** ^**∗**^	−0.24 (0.10)	−0.12 (0.27)
Distance	0.17 (0.19)	0.08 (0.33)	0.18 (0.17)	−0.08 (0.34)	**−0.42 (0.011)** ^**∗**^	−0.21 (0.13)	−0.25 (0.09)
Floors	0.00 (0.50)	−0.16 (0.20)	0.08 (0.33)	−0.15 (0.21)	−0.27 (0.08)	0.25 (0.09)	**−0.42 (0.01)** ^**∗**^
SM	−0.20 (0.14)	**−0.58 (0.001)** ^**∗∗**^	−0.15 (0.22)	0.06 (0.37)	−0.02 (0.46)	0.30 (0.05)	−0.02 (0.45)
LAM	0.13 (0.25)	0.22 (0.12)	**0.39 (0.02)** ^**∗**^	0.02 (0.45)	−0.17 (0.19)	−0.15 (0.22)	0.05 (0.40)
FAM	0.27 (0.08)	0.19 (0.16)	−0.002 (0.50)	−0.12 (0.26)	−0.30 (0.05)	**−0.38 (0.02)** ^**∗**^	−0.02 (0.45)
VAM	0.10 (0.29)	0.18 (0.17)	0.06 (0.38)	**−0.45 (0.01)** ^**∗**^	−0.26 (0.08)	**−0.34 (0.03)** ^**∗**^	−0.07 (0.37)
NC	**0.42 (0.01)** ^**∗**^	0.07 (0.36)	0.23 (0.11)	−0.12 (0.26)	**−0.43 (0.009)** ^**∗∗**^	**−0.47 (0.005)** ^**∗∗**^	−0.04 (0.42)
AC	**0.35 (0.03)** ^**∗**^	0.20 (0.15)	0.27 (0.08)	−0.19 (0.16)	−0.40 (0.015)^**∗**^	**−0.46 (0.006)** ^**∗∗**^	−0.02 (0.45)

*Note:* WBLT, weight‐bearing lunge test; DFS, dorsiflexion strength; EVROM, eversion range of motion; IVROM, inversion range of motion; NC, number of calories burned; *N* = 30. Bold represents < 0.05.

Abbreviations: AC, activity calories; CAIT, cumberland ankle instability tool; FAM, fairly active minutes; LAM, light active minutes; mBESS, modified balance error scoring system; SM, sedentary minutes; VAM, very active minutes.

^∗^ *p* < 0.05.

^∗∗^ *p* < 0.01.

To further explore these relationships, separate backward multiple linear regressions were conducted using variables that were significantly associated with each PA outcomes. All final models were statistically significant (all *p* < 0.05). Significant models were identified for distance walked, floors climbed, sedentary minutes, light active minutes, very active minutes, and total calories burned (all *p* < 0.05). Eversion ROM was negatively associated with distance walked (*B* = −0.40, 95% CI [−0.74, −0.06]) and floors climbed (*B* = −1.15, 95% CI [−2.18, −0.12]). Sedentary minutes were associated with WBLT (*B* = −12.19, 95% CI [−18.80, −5.58]), whereas light active minutes was associated with dorsiflexion strength (% body weight) (*B* = 6.14, 95% CI [0.44, 11.84]). For very active minutes, mBESS (*B* = −0.65, 95% CI [‐1.09, −0.22]) and eversion ROM (*B* = −4.32, 95% CI [‐8.48, −0.16]) were negatively associated with the outcome. In addition, total calories burned was negatively associated with inversion ROM (*B* = −50.39, 95% CI [−96.27, −4.52]) (Table 3). VIFs for all final models ranged from 1.0 to 1.29, indicating no evidence of multicollinearity.

**TABLE 3:** Summary of regression analysis for clinical measurements and physical activity outcomes.

Dependent variable	Predictor	Adjusted *R* ^2^	*B*	*β*	95% CI	*p*
Distance	EVROM	0.146	−0.40	−0.42	**[**−**0.74,** −**0.06]**	**0.021** ^**∗**^

Floor	Side‐hop	0.271	−0.34	−0.31	[−0.72, 0.04]	0.074
	mBESS		−0.09	−0.30	[−0.19, 0.01]	0.086
	EVROM		−1.15	−0.40	**[**−**2.18,** −**0.12]**	**0.030** ^**∗**^
	IVROM		0.32	0.35	[‐0.02, 0.67]	0.063

SM	WBLT	0.314	−12.19	−0.58	**[**−**18.80,** −**5.58]**	**<** **0.001** ^**∗∗**^

LAM	DFS	0.117	6.14	0.39	**[0.44, 11.84]**	**0.036** ^**∗**^

VAM	mBESS	0.263	−0.65	−0.50	**[**−**1.09,** −**0.22]**	**0.004** ^**∗∗**^
	EVROM		−4.32	−0.35	**[**−**8.48,** −**0.16]**	**0.043** ^**∗**^

NC	EVROM	0.262	−134.11	−0.32	[−277.14, 8.92]	0.065
	IVROM		−50.39	−0.38	**[**−**96.27,** −**4.52]**	**0.033** ^**∗**^

*Note:* DFS, dorsiflexion strength; EVROM, eversion range of motion; IVROM, inversion range of motion; *N* = 30. Bold represents < 0.05.

Abbreviations: LAM, light active minutes; mBESS, modified balance error scoring system; SM, sedentary minutes; VAM, very active minutes; WBLT, weight‐bearing lunge test.

^∗^ *p* < 0.05.

^∗∗^ *p* < 0.01.

## 4. Discussion

The purpose of this study was to identify the physical function factors associated with PA in adults with CAI. Through comprehensive analyses, several physical function measures demonstrated associations with different aspect of PA. First, the results indicate a significant association between eversion ROM and distance, suggesting that lower eversion ROM was associated with greater walking distance. Eversion ROM was also negatively associated with floors climbed, suggesting that individuals with lower eversion ROM tended to climb more floors. In addition, the WBLT, a closed‐chain measure of dorsiflexion, demonstrated the strongest association with sedentary minutes, indicating that reduced weight‐bearing dorsiflexion may be associated with greater sedentary behavior. Regarding activity intensity time, dorsiflexion strength was positively associated with light active minutes, suggesting that individuals with greater dorsiflexion strength tended to spend more time engaged in low‐intensity activity. Greater time spent in vigorous activity was associated with better balance and lower eversion ROM. Lastly, inversion ROM demonstrated a significant negative association with total calories burned, suggesting that individuals with greater inversion ROM tended to expend fewer calories throughout daily activity.

Dorsiflexion strength, inversion and eversion ROM, balance, and WBLT were associated with different aspects of PA. Understanding these factors may help inform rehabilitation strategies aimed at improving PA levels and promoting overall health in individuals with CAI.

### 4.1. ROM

Our results demonstrated that eversion ROM was negatively associated with both distance walked and floors climbed. In addition, inversion ROM was negatively associated with total calories burned. Biomechanical studies may provide insight into inversion and eversion ROM changes during a variety of movements and activities. Previous studies reported that individuals with CAI displayed significantly inverted foot position during the entire gait cycle compared to the healthy control [24]. This altered alignment may be associated with an increased likelihood of giving‐way episodes or recurrent ankle sprain and may indicate changes in movement patterns during daily activities.

In addition to inversion and eversion ROM, reduced WBLT was associated with greater sedentary time. The WBLT has been shown to be associated with dynamic balance in individuals with CAI. Reduced weight‐bearing dorsiflexion ROM may limit the ability to achieve the close‐packed position, which is the most stable position of the ankle during functional movement [25], and this limitation may be a risk factor for recurrent ankle sprains [26]. Moreover, dorsiflexion ROM deficits have been found to lead to altered movement strategies, specifically in the kinematics of the knee and hip joints during the landing phase [25, 27]. Therefore, this may suggest that deficits in weight‐bearing dorsiflexion ROM may impact ankle function and may be related to increased sedentary time.

Another explanation may be due to longitudinal changes in tissue mechanics. Ligamentous mechanical ankle laxity refers to an increased ROM that exceeds the typical physiological limits, which can be attributed to alterations in ligamentous tissue or changes in arthrokinematics [28, 29]. However, ROM alone does not imply mechanical impairment since the presence of mechanical laxity in the ankle can be identified by excessive inversion of rearfoot observed through an instrumented arthrometer or manual stress test [30, 31].

### 4.2. Strength

Previous studies reported significant ankle strength deficits in individuals with CAI [6, 32–34]. In this study, greater dorsiflexion strength was associated with longer durations of light‐intensity activities, suggesting that ankle dorsiflexors may play a role in sustaining movement during daily activities. Consistent with these findings, prior studies have demonstrated dorsiflexion strength deficits in individuals with CAI [35, 36]. These findings suggest that dorsiflexion strength deficits may be associated with lower PA behavior in individuals with CAI. Although few studies have investigated this relationship, our results suggest that strengthening ankle dorsiflexor muscles may help support ankle stability and PA participation in individuals with CAI. Future research should explore whether ankle strengthening intervention can directly increase PA participation in individuals with CAI.

### 4.3. Balance

We found that better balance was associated with increased time spent in vigorous activity. Extensive research has consistently highlighted the presence of balance deficits in individuals with CAI [37]. Doherty et al. documented poor dynamic balance among CAI patients [38, 39], and Zhang et al. found poor static and dynamic balance performance in this population [40]. The impaired postural control resulting from ligamentous injury has been recognized as a major contributing factor to the limitations in stability and perceived function experienced by patients with CAI [41]. Proper postural control is an essential requirement for any form of PA [42]. Additionally, the significance of balance training for individuals with CAI has been underscored in numerous published studies as well [43]. Therefore, our findings further support the importance of improving balance to facilitate active engagement in physical activities for individuals with CAI.

### 4.4. Limitations

Several limitations should be acknowledged. First, ankle laxity was not directly assessed via arthrometer, and ankle ROM alone may not fully capture mechanical instability in individuals with CAI [28, 44]. Further studies should include objective measures of mechanical laxity and examine its relationship with PA outcomes.

Second, although 30 individuals with CAI are a respectable sample size for this pathologic patient population, a larger sample would improve the ability to perform multifactorial regressions. Moreover, most participants were recruited from a college setting and included recreational or competitive athletes, which may limit the generalizability.

Third, the wearable device used in this study did not provide detailed information on daily wear time. Although nonwear days could be identified, partial nonwear within a day could not be determined, and activities performed during these periods were not captured.

Additionally, calorie‐based outcomes may be influenced by body mass. Supplementary analyses including body mass as a covariate for the number of calories burned showed that body mass remained the only significant predictor, and previously observed association was no longer significant. Specifically, body mass was significantly associated with total calories burned (*B* = 39.38; adjusted *R* ^2^ = 0.686; 95% CI [29.32, 49.45], Table 4). Furthermore, multiple bivariate analyses were conducted without adjustment for multiple comparisons, and variables were selected based on these results, which may increase the risk of type I error and selection bias. Therefore, findings should be interpreted as exploratory.

**TABLE 4:** Supplementary regression analyses including body mass as a covariate.

Dependent variable	Predictor	Adjusted *R* ^2^	*B*	*β*	95% CI	*p*
NC	Body mass	0.686	39.38	0.84	[29.32, 49.45]	< 0.001

*Note:* NC, number of calories burned.

## 5. Conclusion

Our findings of this study suggest that selected physical function variables, including eversion ROM, inversion ROM, WBLT, balance, and dorsiflexion strength, may be associated with aspects of PA in individuals with CAI. These findings provide preliminary insight into potential factors related to PA. However, future studies with larger sample sizes are needed to confirm these findings and to better understand the relationship between physical function and PA in individuals with CAI.

## Funding

This work was supported by a 2022 University of Nebraska at Omaha’s Graduate Research and Creative Activity grant.

## Conflicts of Interest

The authors declare no conflicts of interest.

## Data Availability

The data that support the findings of this study are available from the corresponding author upon reasonable request.
